# Acute scrotal swelling following perforated rectal carcinoma with abscess formation

**DOI:** 10.1259/bjrcr.20150284

**Published:** 2016-11-02

**Authors:** Vishnusai Chauhan, Matthew Newman, Rakesh Sinha

**Affiliations:** ^1^Department of Colorectal Surgery, Warwick Hospital, South Warwickshire Foundation Trust, Warwick, UK; ^2^Department of Radiology, Warwick Hospital, South Warwickshire Foundation Trust, Warwick, UK

## Abstract

A 59-year-old cachectic male was referred to the surgical outpatient department with intermittent haematochezia and a longstanding change in bowel habit with associated weight loss and anaemia. Following investigation, he was diagnosed with a large rectal tumour with multiple metastases. 7 days later, the patient presented again with fevers, bilious vomiting, abdominal pain and distension. On examination, he had a generally tender abdomen,= although no peritonism, but an enlarged, extremely tender hemiscrotum with no cough reflex. Imaging revealed a perforated rectum and subsequent abscess formation, which tracked *via* an unusual anatomical route to present as scrotal swelling.

## Clinical presentation

**Figure 1:**
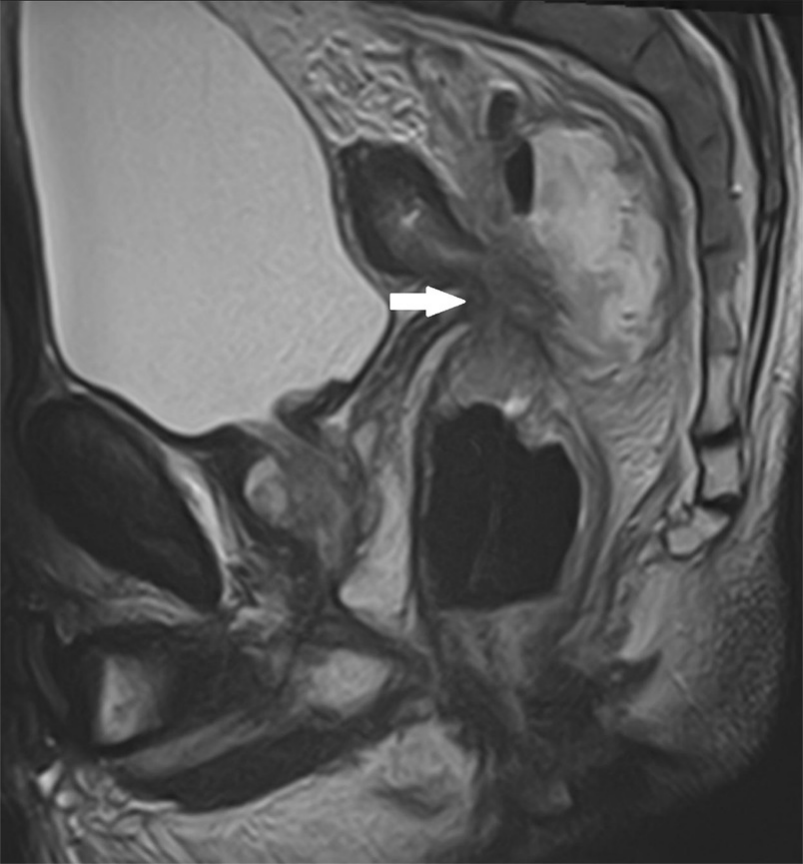
Sagittal *T*_2_ weighted MRI: arrow shows rectal tumour with an abscess posterior to the tumour.

**Figure 2.**
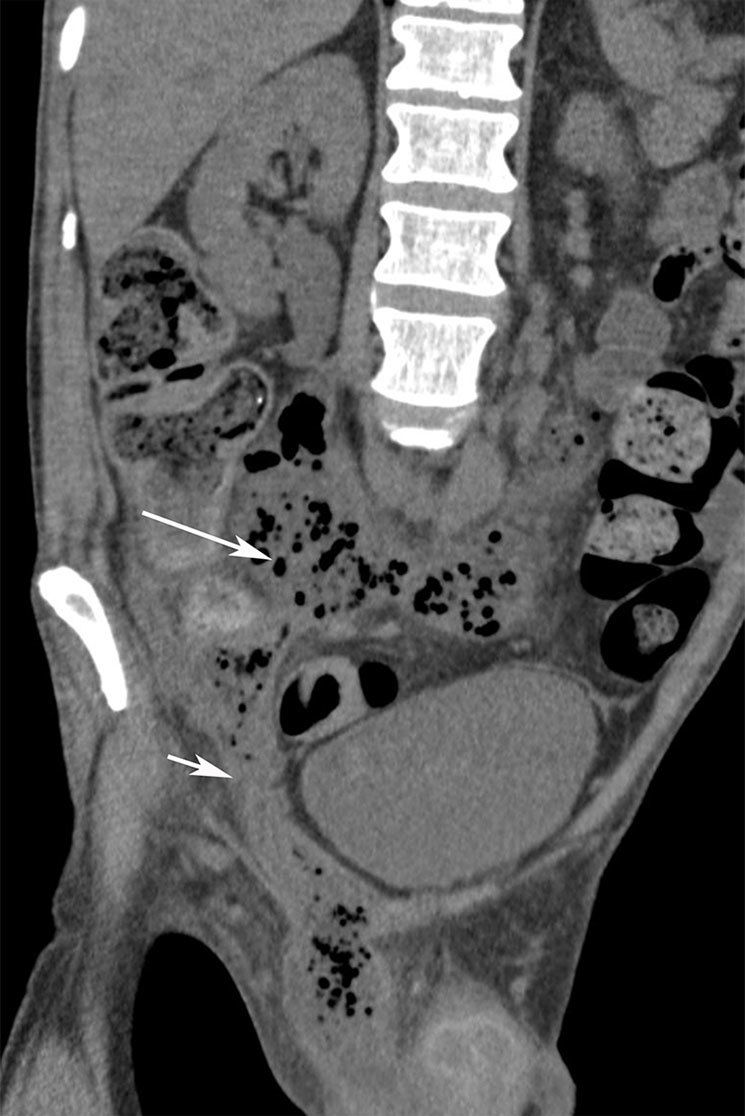
Coronal oblique venous phase contrast-enhanced CT: long arrow points to a large retroperitoneal abscess while the short arrow points to the tracking *via* the obturator space into the scrotum.

A 59-year-old cachectic male was referred to the surgical outpatient department with intermittent haematochezia and a longstanding change in bowel habit with associated weight loss and anaemia. He underwent flexible sigmoidoscopy, which showed a large rectal tumour. A staging CT scan confirmed malignancy in the rectosigmoid region with extensive metastases to the liver and spleen together with evidence of para-aortic and mesenteric lymphadenopathy. There was also a loculated collection seen just posterior to the tumour on MRI performed for local staging (Figure 1). A multidisciplinary decision was made for a planned admission in 10 days for performing a defunctioning colostomy and palliative radiotherapy. However, 7 days later, the patient presented with fever, bilious vomiting, abdominal pain and distension. On examination, he had a generally tender abdomen, although there was no peritonism. He also had a red, exquisitely tender right hemiscrotum with a mass that was not palpable above and had no cough impulse. His white cell count was 32.1 × 109 l^−1^, C-reactive protein was 259 mg l^−1^ and haemoglobin was 102 g l^−1^. A repeat CT scan was ordered to investigate the cause of the scrotal swelling (Figure 2).

## Imaging findings

**Figure 3.**
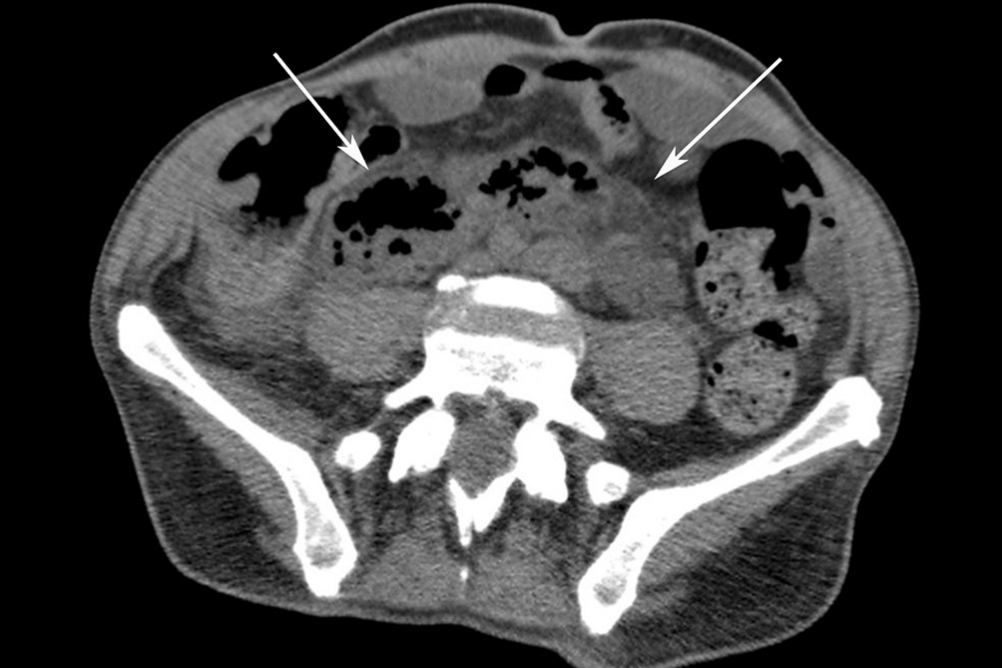
Axial venous phase contrast-enhanced CT image showing a large retroperitoneal abscess containing air pockets (arrows).

**Figure 4.**
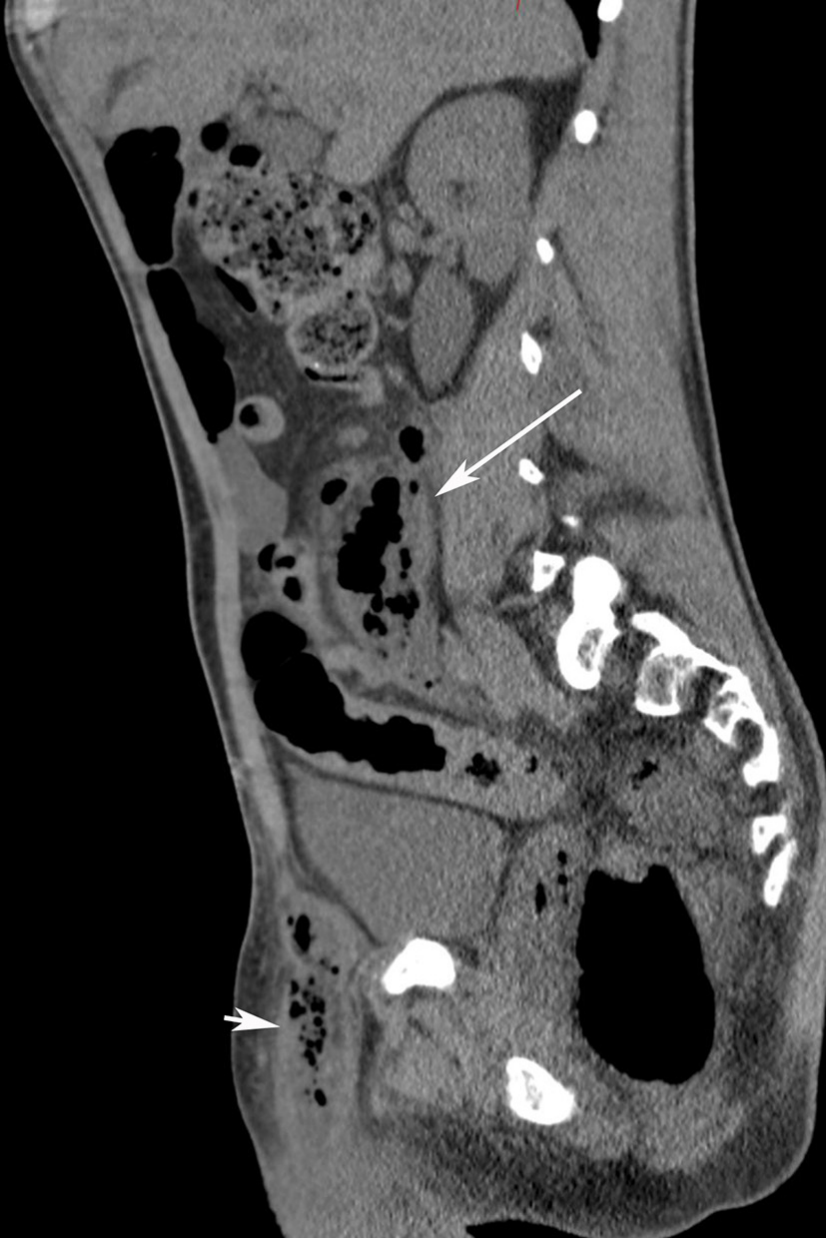
Sagittal venous phase contrast-enhanced CT scan showing the retroperitoneal abscess tracking up from the pelvis (long arrow) and an inguinal abscess (short arrow).

The abscess, in this case, tracked bilaterally in the retroperitoneal space up to the retropancreatic region (Figures 2 and 3). The abscess also progressed along the right iliac and obturator spaces to present in the right inguinal canal as a scrotal abscess (Figures 2 and 4).

An alternative pathway for an abscess to form in the rectal region and present in the right testicle would have been through the peritoneum and inferiorly towards the pelvic cavity through the inguinal canal. However, in such cases the symptoms are usually different, with evidence of peritonitis.

## Differential diagnosis

Differentials for the clinical presentation would include scrotal abscess, acute testicular torsion, strangulated right inguinoscrotal hernia and Fournier’s gangrene. However, given the prior knowledge of the patient's tumour and imaging, the last three were much less likely.

## Treatment

He was initially resuscitated with fluids and given targeted antibiotics as per microbiological guidance. Owing to his large tumour and abscess, it was felt that surgical management would be more appropriate than attempting image-guided drainage. The patient underwent a laparotomy with insertion of a retroperitoneal and pelvic drains to treat the abscess; in addition, an end colostomy was also fashioned with a palliative intent. He remained an inpatient for 4 more weeks and was discharged home with Macmillan nurse follow-up.

## Discussion

Colorectal tumour perforations usually present with intraperitoneal collections leading to a clinical presentation of vague abdominal pain and septicaemia. Colorectal tumour perforation is thought to result from tumour necrosis or diastatic perforation, with the incidence ranging from 2.6% to 9%.^1^ There does not seem to be a strong consensus between tumour stage and perforation;^1^ however, there is evidence that some chemotherapy agents such as bevacizumab can increase the risk of bowel perforation and have an associated poor outcome.^2^ Fractionated radiotherapy may also have a role in increasing the risk of perforation of the bowel malignancy. The most common cause of colorectal perforation overall is diverticular disease, with ischaemic colitis and iatrogenic injury being more likely causes over malignancy. Trauma and deep ulcers are less probable.^3^ Clinical manifestations include abdominal pain, fever, rigors and sepsis. The associated mortality depends on a variety of features such as age, presence of pre-operative sepsis and patient comorbidities.^3^

Scrotal swelling as a result of abdominal abscesses is unusual. They may occur more frequently in children, following perforated appendicitis or appendectomies, owing to a patent processus vaginalis.^4^ Patent processus vaginalis is present in approximately 80–90% of newborns, but declines to 15–37% in adults; thus scrotal abscesses are less common in adults.^4^

Other causes of scrotal swelling include Fournier’s gangrene, strangulated or obstructed inguinal hernias, hydrocele, varicocele, spermatocele, localized oedema from insect bites, nephrotic syndrome, testicular torsion and testicular cancer.^5^

Intraperitoneal abscesses are associated with high morbidity and mortality and thus require urgent intervention by image-guided drainage or operative management.

## Learning points

1. Tumour perforation can complicate up to 9.6% of colorectal cancers, with necrosis being the main pathological cause and diastatic perforation occurring less commonly.
2. A patent processus vaginalis can lead to an inguinoscrotal abscess from an intra-abdominal pathology, more commonly in children; however, in adults, this may occur *via* tracking through the subperitoneal and extraperitoneal spaces into the inguinal canal. This case highlights that the obturator space may also provide an alternative pathway for an intra-abdominal abscess to progress into the scrotum.
3. Important differentials of acute scrotal swelling include Fournier’s gangrene, testicular torsion and acute inguinal hernias.

## Consent

Written consent was obtained.
